# Profiling Analysis of N6-Methyladenosine mRNA Methylation Reveals Differential m6A Patterns during the Embryonic Skeletal Muscle Development of Ducks

**DOI:** 10.3390/ani12192593

**Published:** 2022-09-28

**Authors:** Biao Chen, Shuibing Liu, Wentao Zhang, Ting Xiong, Mingfang Zhou, Xiaolong Hu, Huirong Mao, Sanfeng Liu

**Affiliations:** 1 College of Animal Science and Technology, Jiangxi Agricultural University, Nanchang 330045, China; 2 Poultry Institute of Jiangxi Agricultural University, Nanchang 330045, China

**Keywords:** duck, embryonic muscle development, breast muscle, N6-methyladenosine methylation, MeRIP-seq

## Abstract

**Simple Summary:**

Recent studies show that N6-methyladenosine (m6A) modification, the most common RNA chemical modification, influences the modification, processing, transport, and translation of RNA. N6-methyladenosine is an epigenetic modification that influences skeletal myogenesis and skeletal muscle development. However, the N6-methyladenosine modification profile and its function during poultry muscle development is unclear, and there is only one report about m6A modification in ducks, which focuses on duck hepatitis A virus infection. Here, we compared the m6A modification profiles between E13 (embryonic day 13) and E19 (embryonic day 19) in duck breast muscle differentiation using MeRIP-seq, and evaluated the expression profile of the methyl transferase METTL14 and its cofactors during breast muscle development. This is the first study of N6-methyladenosine modification patterns in duck muscle tissue. The current study not only elucidates the regulation mechanisms of duck skeletal muscle development, but also lays the groundwork for studying the role of RNA modification in poultry muscle development.

**Abstract:**

N6-Methyladenosine is a reversible epigenetic modification that influences muscle development. However, the m6A modification profile during poultry skeletal muscle development is poorly understood. Here, we utilized m6A-specific methylated RNA immunoprecipitation sequencing to identify m6A sites during two stages of breast muscle development in ducks: embryonic days 13 (E13) and E19. MeRIP-seq detected 19,024 and 18,081 m6A peaks in the E13 and E19 groups, respectively. Similarly to m6A distribution in mammalian transcripts, our results revealed GGACU as the main m6A motif in duck breast muscle; they also revealed that m6A peaks are mainly enriched near the stop codons. In addition, motif sequence analysis and gene expression analysis demonstrated that m6A modification in duck embryo skeletal muscles may be mediated by the methyltransferase-like 14. GO and KEGG analysis showed that m6A peaks containing genes at E19 were mainly enriched in muscle-differentiation- and muscle-growth-related pathways, whereas m6A peaks containing genes in E13 were mainly enriched in embryonic development and cell proliferation pathways. Combined analysis of MeRIP-seq and RNA-seq showed that the mRNA expression may be affected by m6A modification. Moreover, qRT-PCR analysis of the expression of *METTL14* and its cofactors (*WTAP*, *ZC3H13*, *RBM15* and *VIRMA*) during duck embryonic skeletal muscle development in breast and leg muscle samples revealed a significant downward trend as the developmental age progressed. Our results demonstrated that m6A mRNA methylation modifications control muscle development in ducks. This is the first study of m6A modification patterns in duck muscle tissue development, and it lays the foundation for the study of the effects of RNA modification on poultry skeletal muscle development.

## 1. Introduction

Poultry meat contributes to human nutrition by providing high-quality protein [1]. Poultry muscle development is a complex process which is influenced by many factors, such as genetics, nutrition, and disease [2,3,4]. However, genetics plays the main role [5,6]. Myogenesis occurs in the following stages: the differentiation of somites into myoblasts, the migration of myoblasts to the limb buds and the expression of specific myogenic transcription factors, and the differentiation of myoblasts into myotubes under the influence of these myogenic transcription factors [7]. The transition point from the proliferation to the fusion of duck embryonic breast muscle development occurs at embryonic day 19 (E19), and this coincides with the highest expression level of myogenic markers, such as myogenin, MRF4, and myostatin [8].

RNA modification has emerged as a research hotspot in recent years [9]. Recent studies show that modification of N6-methyladenosine (m6A), the most common RNA chemical modification, influences the modification, processing, transport, and translation of RNA [10]. m6A occurrence and function are mainly driven by methyltransferases (also known as writers), demethylases (also known as erasers), and methyl recognition proteins (also known as readers) [10]. m6A methyltransferase methylates adenine on RNA [11]. The main methyltransferases are methyltransferase-like 3 (METTL3) and METTL14. The METTL3/METTL14 regulatory cofactors, including WT1-related protein (WTAP), RNA-binding element protein 15 (RBM15), and zinc finger CCCH-type containing 13 (ZC3H13) [11,12,13,14]. m6A demethylases, including FTO α-ketoglutarate dependent dioxygenase (FTO)) and AlkB homologue 5 (ALKBH5), catalyze RNA m6A demethylation. m6A methyl recognition protein can read RNA methylation modification and influence the processing and function of subsequent RNA, including alternative mRNA splicing, secondary structure transformation, nucleation, stabilization, degradation, and translation [15,16]. m6A methyl recognition proteins include YTH-domain-containing (YTHDC) proteins 1 and 2, YTH N6-methyladenosine RNA-binding proteins 1-3 (YTHDF1-3), and insulin-like growth factor 2 mRNA-binding protein 1-3 (IGF2BP1-3) [17].

Numerous studies have investigated the regulatory role of m6A during muscle development [18]. Although m6A plays an important role in livestock growth and development [18,19,20,21,22], its role in poultry muscle development is still unknown. The purpose of the current study was to profile the mRNA m6A modification at different stages in duck breast muscle development. Here, undifferentiated (E13) and differentiated (E19) duck breast muscles were collected and subjected to MeRIP-seq and mRNA-seq analysis to profile m6A modification sites during duck breast muscle development. The expression levels of *METTL14*, *WTAP*, *ZC3H13*, *RBM15*, and *VIRMA* were determined at different stages during breast and leg muscles development. The data generated from this study can be utilized in future studies of m6A function in poultry skeletal muscle development.

## 2. Materials and Methods

### 2.1. Ethics Statement

All animals used in this experiment adhered to the ethical guidelines of Jiangxi Agricultural University (JXAULL-2017002). The approval code for the current experiment is JXAU#2021D002. All embryos and ducklings were sacrificed humanely.

### 2.2. Animals and Tissue Collection

A total of 180 fertilized eggs from 240-day-old Shanma ducks were purchased from the Jiangxi Tianyun agricultural development company (Nanchang, China). The eggs were stored at 18 °C for one day, and all eggs and the incubator were fumigated using 21 g/m^3^ KMnO_4_ and 42 mL/m^3^ formalin before the incubation. All eggs were incubated at 37.8 °C in a fully automatic egg incubator (JT35, Jitan, China) with 60% relative humidity for 24 days. Then, all eggs were transferred to the hatcher tray from the 25th day to the 28th day in the same incubator, with a temperature of 36.9 °C and 70% relative humidity. All eggs were candled at the 6th day and the 25th day and dead embryos were removed from the incubator. The interval for egg turning was set at 2 h during the incubation, and the egg-turning was halted after all eggs were placed in the hatcher tray. Tissue collection was conducted as described in our previous work [23]. Breast muscles and leg muscles from 15 embryos were collected every 3 days from embryonic day 10 (E10) to 1 day post hatch (E10, E13, E16, E19, E22, and 1 day post hatch). The embryos were carefully taken out and their developmental stages were confirmed by morphological observation. Next, breast muscles and leg muscles were sampled, snap-frozen in liquid nitrogen, and stored at −80 °C. Total DNA from duck embryo breast muscles was extracted using a Trelief^TM^ Animal Genomic DNA Kit (Tsingke, Beijing, China) following the manufacturer’s instructions. The sex of the embryos was determined by PCR amplification analysis using sex-specific primers of chromodomain helicase DNA-binding protein 1 (*CHD1*) gene [24]. A total of 6, 9, 5, 6, and 7 female embryos and 9 female ducklings were identified from E10, E13, E16, E19, E22 and 1 day post hatch (P1), respectively. Five females from each period were selected and used in this study.

### 2.3. RNA Extraction and m6A-Specific Methylated RNA Immunoprecipitation (MeRIP)

Total RNA was extracted from the breast muscle and leg muscle tissues of E10, E13, E16, E19, E22, and P1 ducks using TRIzol reagent (Invitrogen, CA, USA), following the manufacturer’s instructions. Three breast muscle samples of E13 and E19 were chosen for MeRIP-seq. To remove any DNA contamination from the samples, DNase I was used after RNA extraction. RNA quality was determined on Nanodrop 1000 (NanoDrop, Wilmington, DE, USA). RNA integrity was confirmed by 1.5% agarose gel electrophoresis and quantified by Qubit3.0 using a QubitTM RNA Broad Range assay kit (Life Technologies, Carlsbad, CA, USA). In total, 50 μg of total RNA was used for polyadenylated RNA enrichment using VAHTS mRNA capture beads (Vazyme, Nanjing, China). Then, 20 mM ZnCl_2_ was added to the mRNA and incubated at 95 °C for 10 min to generate 100–200 nt RNA fragments. Next, 10% of the RNA fragments were saved as “input” and the rest were used for m6A immunoprecipitation (IP) using a specific anti-m6A antibody (Synaptic Systems, Göttingen, Germany). The IP experiment was carried out according to the protocol described in previous research [25].

### 2.4. Library Constructions and Sequencing

For MeRIP-seq, the stranded RNA sequencing library was constructed using a KC-DigitalTM Stranded mRNA Library Prep kit from Illumina^®^ (Seqhealth, Wuhan, China), following the manufacturer’s instructions. The kit eliminates duplication bias in the PCR and sequencing steps by using a unique molecular identifier (UMI) of 8 random bases to label pre-amplified cDNA. The library products were enriched, quantified, and sequenced on a Novaseq 6000 sequencer (Illumina, San Diego, CA, USA) with pair-end 150.

To analyze mRNA expression, in addition to the RNA-seq with input, total RNAs were used to construct a library of 200–500 nt RNA fragments to enhance the accuracy of gene expression results. Then, 2 μg of RNA were used for stranded RNA sequencing library preparation, using a protocol similar to the one used for MeRIP-seq, except for 200–500 nt library products. All sequencing data in this study can be found in the NCBI Sequence Read Archive (SRA) (https://submit.ncbi.nlm.nih.gov/, accessed on 22 September 2022) with the accession numbers PRJNA725663 (MeRIP-seq data) and PRJNA726590 (RNA-seq data).

### 2.5. MeRIP-seq and RNA-seq Data Analysis

For MeRIP-seq: first, raw sequencing data were filtered using Trimmomatic [26]. Low-quality reads were then discarded and reads contaminated with adaptor sequences were trimmed. Clean reads were further treated to minimize duplication. In brief, clean reads were first clustered according to the UMI sequence, whereby reads with the same UMI sequence were grouped into the same cluster. Reads in the same cluster were compared using pairwise alignment and reads with sequence identities > 95% were extracted to a new sub-cluster. After all sub-clusters were generated, multiple sequence alignment was used to obtain one consensus sequence for each sub-cluster.

The de-duplicated consensus sequences were used for m6A peak analysis. They were mapped to NCBI’s duck (Anas platyrhynchos) reference genome (https://www.ncbi.nlm.nih.gov/assembly/GCF_003850225.1/, accessed on 22 September 2022) using STAR software (Version 2.5.3a) with default parameters. ExomePeak version 3.8 was used for peak calling. The m6A peaks were annotated using bedtools version 2.25. DeepTools version 2.4.1 was used for peak distribution analysis. Next, peaks present in both groups were classified as intersection peaks, and group-unique peaks were classified as specific peaks. Differential m6A peaks were identified in the intersection peaks using Fisher’s test with *p* < 0.05 and |log2FC| > 1 as the cutoff threshold. Sequence motifs enriched in m6A peak regions were identified using Homer version 4.10. Gene Ontology (GO) and the Kyoto Encyclopedia of Genes and Genomes (KEGG) enrichment analyses for annotated genes were carried out using KOBAS version: 2.1.1 with a corrected *p* value cutoff of 0.05 to judge statistically significant enrichment. Protein–protein interaction analysis was carried out on STRING version 11.0 and adjusted on Cytoscape version 3.7.2).

For RNA-seq analysis, raw data and reads mapping were processed same as for MeRIP-seq. Reads mapped to exons were summarized by featureCounts (Subread-1.5.1; Bioconductor) and reads per kilobase per million mapped reads (RPKM) were calculated. Differentially expressed genes (DEGs) in E19 vs. E13 were identified using edge R package (version 3.12.1) using *p* < 0.05 and |log2FC| > 1 as cutoffs. Enrichment and protein–protein interaction analyses were conducted using the procedure used for MeRIP-seq. For Pearson correlation analysis of mRNA expression and m6A abundance, RPKM and m6A enrichment were log2 transformed and m6A enrichment was calculated by dividing IP RPKM by input RPKM.

### 2.6. qRT-PCR Assay

Total RNAs were reverse transcribed using the Monad MonScript™ All-in-One Kit with DNase (Biopro, Shanghai, China). The expression level was determined using 2 × T5 Fast qPCR Mix (TsingKe, Beijing, China) in a final volume of 20 µL. *GAPDH* served as the internal control. The qRT-PCR reaction was carried out on an ABI QuantStudio 5 system (Thermo Fisher, Waltham, MA, USA) under the following protocol: 95 °C for 3 min; 40 cycles of 95 °C for 10 s, Tm for 30 s min, and fluorescence collection at 65–95 °C. Each sample was analyzed in triplicate and the relative mRNA expression level was calculated using the 2^−∆∆Ct^ method. The qRT-PCR primers’ sequences are included in Table S1.

The qPCR results are presented as the mean ± standard error of the mean (S.E.M.). For multiple comparison analysis, groups were compared with a one-way ANOVA test followed by a Duncan test using SPSS 26.0 (https://www.ibm.com/support/pages/downloading-ibm-spss-statistics-26, accessed on 22 September 2022). The different letters between the two groups represent significant differences (*p* < 0.05). For two-group comparison, the results were subjected to statistical analysis using the two-tailed student’s *t*-test. The level of significance was presented as * (*p* < 0.05), ** (*p* < 0.01), and *** (*p* < 0.001).

## 3. Results

### 3.1. Transcriptome-Wide Detection of m6A Peaks in Duck Embryonic Breast Muscle

MeRIP-seq analysis of six embryonic breast muscle tissues generated 91.7 Gb in data (1,426,191,010 raw reads). After data quality control, 672,172,477 clean reads were aligned to the duck reference genome. This analysis revealed a unique mapping ratio of 83.46 to 88.22% (Table 1). RNA-seq analysis of the same samples used in MeRIP-seq produced 38.6 Gb of data and a total of 429,253,704 reads were mapped to the reference genome with a unique mapping range of 82.78–88.06% (Table 2). Furthermore, the correlation coefficient value of MeRIP-seq samples of the same group was high (about 0.97) (Figure S1).

**Figure 1:**
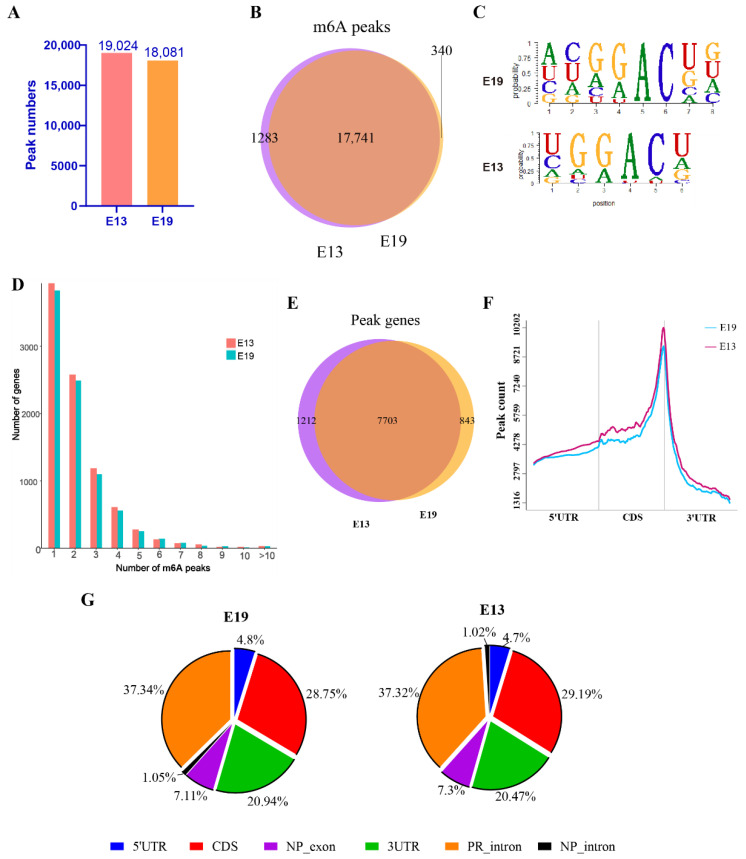
Analysis of transcriptome-wide MeRIP-seq data and m6A peaks. (**A**) Number of m6A peaks in MeRIP-seq data of E13 and E19. (**B**) Venn diagram showing m6A peaks found in E19 and E13. (**C**) Top m6A consensus motifs identified in all m6A peaks. (**D**) Number of m6A peaks in each gene. (**E**) Venn diagram showing the peak genes identified in E19 and E13. (**F**) Abundance of m6A peaks per a mRNA. (**G**) The distribution of m6A peaks on different regions of a gene. NP exon refers to exon on the non-coding gene; PR intron indicates intron on the coding gene; NP intron indicates intron of the non-coding gene.

MeRIP-seq identified 19,024 m6A peaks in E13 breast muscle tissues and 18,081 m6A peaks in E19 group (Figure 1A), with 17,741 peaks common to the two groups, 1283 peaks unique to E13, and 340 peaks unique to E19 (Figure 1B). Using homer to scan motifs between peaks, we identified GGACU as the most likely motif sequence in duck embryo breast muscle tissue (Figure 1C). A count of each gene’s peak number found that >73% of the mRNAs had 1–2 peaks, and about 13% of the genes had ≥4 m6A peaks in both groups (Figure 1D and Figure S2). Counting the number of m6A peak-associated genes in each group, we detected 8915 m6A peak genes in E13 tissue and 8546 m6A peak genes in E19 samples. Comparison of the two sets of genes identified 7703 intersection genes (Table S2), while 1212 and 843 genes were specific to E13 and E19, respectively (Figure 1E, Tables S3 and S4). Information about the functional regions of genes, where all peaks were located, can be determined by peak location. In embryonic duck breast muscle, m6A peaks were mainly distributed in the anterior and posterior regions of the stop codon (the CDS and 3′UTR regions close to the stop codon, Figure 1F). According to annotation and statistics, the distribution of peaks in different regions were introns beyond the coding gene (PR intron, about 37%), CDS region (about 29%), and 3′UTR region (about 20%, Figure 1G).

### 3.2. GO and KEEG Pathway Analysis of Differentially m6A-Methylated mRNAs in E13 and E19

**Figure 2:**
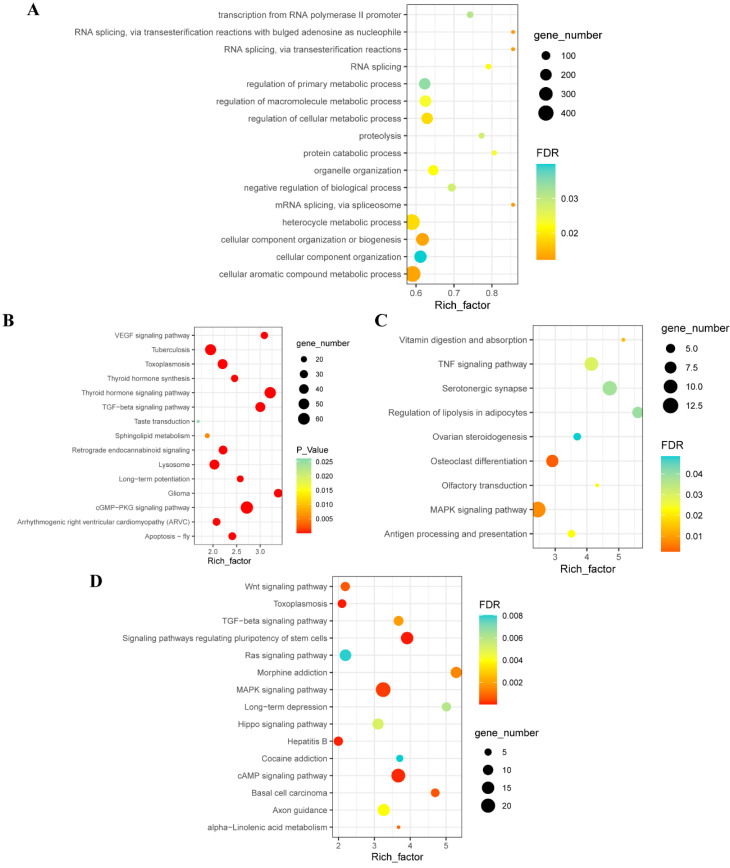
GO biological process and KEGG pathway analysis of group-specific and intersecting m6A-containing genes. (**A**) GO biological process enrichment analysis of the intersection of m6A peak genes and gene expression levels. GO terms with FDR < 0.05 are shown. (**B**) KEGG enrichment analysis of the intersection of m6A peak genes and gene expression levels. The top 15 pathways are shown. (**C**) KEGG enrichment analysis of E19-m6A-peak-associated genes. Pathways with FDR < 0.05 are shown. (**D**) KEGG enrichment analysis of E13-m6A-peak-associated genes. The top 15 pathways are shown.

To explore the important biological roles and pathways m6A actively participates in during the embryonic development of duck breast muscle, we subjected differentially m6A-methylated mRNAs in E13 and E19 to GO and KEGG enrichment analysis. GO biological process (BP) analysis of all the differentially expressed genes revealed that the m6A-containing genes are significantly enriched in RNA splicing, cell metabolism, and cell component organization (Figure 2A, Table S5). In addition, the KEGG pathway revealed m6A-containing genes to be significantly involved in TGF-beta, VEGF, and cell proliferation and embryonic development pathways (Figure 2B, Table S6). In E19, the KEGG enrichment analysis found that m6A-containing genes were significantly enriched in MAPK and TNF signaling (Figure 2C, Table S7), while in E13, the m6A-containing genes were mainly enriched in signaling pathways regulating stem cell pluripotency, cAMP signaling, and signal pathways involved in cell pluripotency and proliferation (Figure 2D, Table S8).

**Figure 3:**
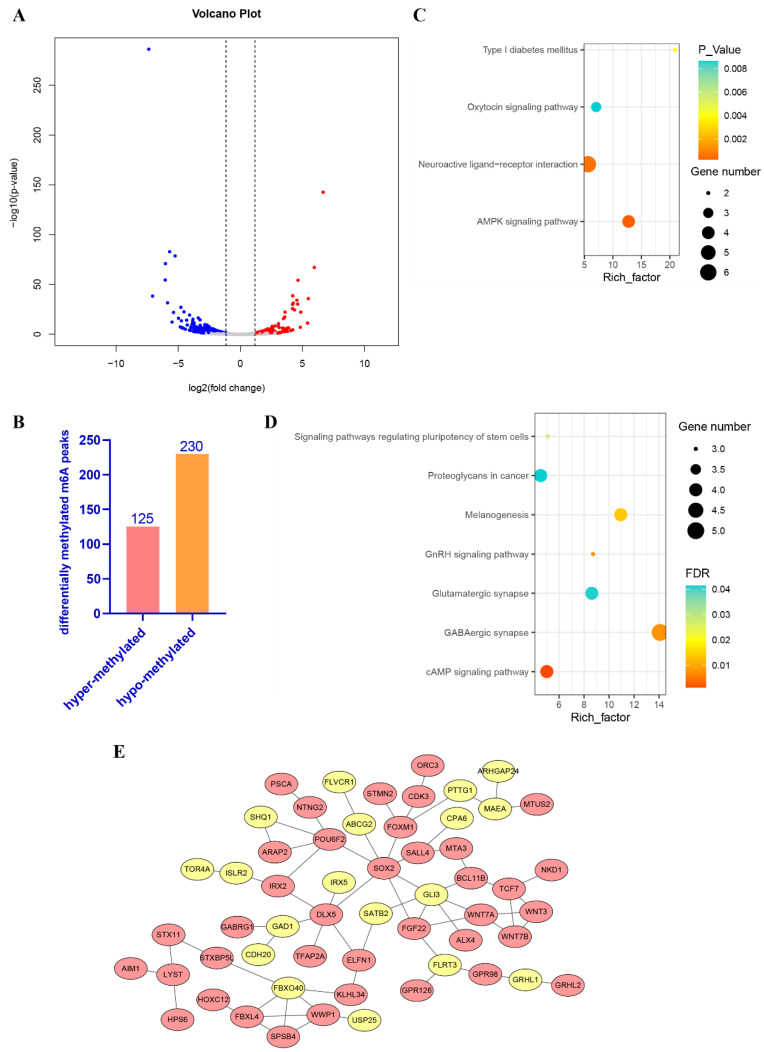
The dynamic changes in m6A modification in E13 and E19. (**A**) Volcano charts showing abundance of the intersection m6A peaks in E19 and E13. The differentially methylated peaks were chosen based on a *p* value of < 0.05 and |log2FC| > 1. (**B**) The number of differentially methylated peaks in intersection peaks in E19 and E13. (**C**) KEGG enrichment analysis of the hyper-methylated peak genes in E19 and E13. The pathways with *p* value < 0.01 are shown. (**D**) KEGG enrichment analysis of the hypo-methylated peak genes in E19 and E13. The pathways with FDR < 0.05 are shown. (**E**) The protein–protein interaction network of differentially methylated peak genes. Yellow nodes stand for the hyper-methylated peak genes; the pink nodes indicate the hypo-methylated peak genes.

To investigate the dynamic changes in m6A modification at different stages (E19 vs. E13) of embryonic duck skeletal muscle development, the abundance of m6A modification at intersection peaks between E13 and E19 were compared. Thresholds of *p* value < 0.05 and |log2FC| > 1 were used to identify differentially methylated peaks (DMPs). Differentially methylated genes (DMGs), which are genes associated with DMPs, were submitted to the enrichment analysis. Based on the threshold, 355 m6A peaks were differentially abundant in the 17,741 intersection peaks in E13 and E19 samples (Figure 3A, Table S9). The DMPs had 230 peaks that were significantly hypo-methylated and 125 peaks that were noticeably hyper-methylated (Figure 3B). DMPs annotation identified 331 DMGs (Table S10). KEGG pathway enrichment analysis of the DMGs revealed that the hyper-methylated peak genes were significantly enriched in 21 pathways, including the AMPK signaling pathway and the neuroactive ligand-receptor interaction pathway (Figure 3C, Table S11). The hypo-methylated peak genes were significantly enriched in the pathways related to cell proliferation and growth, including cAMP and GnRH signaling (Figure 3D, Table S12). Protein–protein interaction network analysis of DMGs on STRING identified SRY-Box Transcription Factor 2 (SOX2), Distal-Less Homeobox 5 (DLX5), and F-Box Protein 40 (FBXO40), which are involved in myogenesis and muscle growth, as central factors in the network (Figure 3E).

### 3.3. Identification of Differentially Expressed Genes (DEGs) in E19 vs. E13 Samples

**Figure 4:**
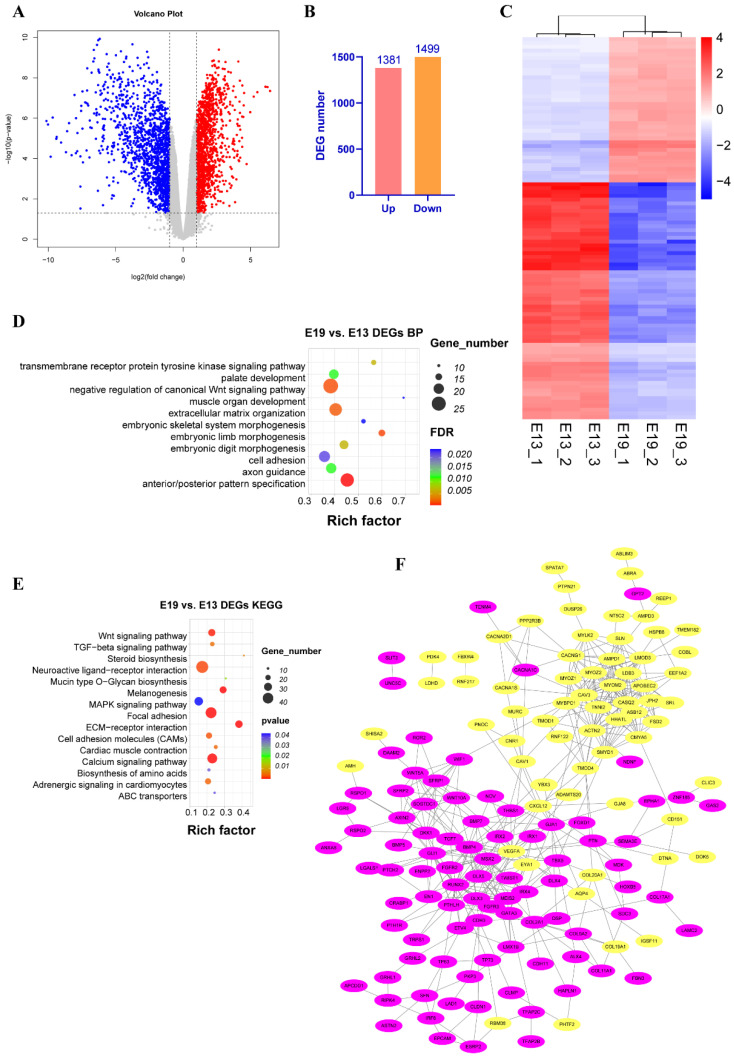
Identification of the DEGs in E19 and E13 by RNA-seq. (**A**) Volcano charts of the differentially expressed genes in E19 and E13. The genes were chosen based on a *p* value of < 0.05 and |log2FC| > 1 criteria. (**B**) The number of the DEGs in E19 and E13. (**C**) Heat maps of the DEGs in E19 and E13. The top 100 DEGs are shown. (**D**) GO biological process enrichment analysis of the DEGs. The terms with FDR < 0.05 are shown. (**E**) KEGG enrichment analysis of the DEGs in E19 and E13. The top 15 pathways are shown. (**F**) The protein–protein interaction network of the DEGs in E19 and E13. Yellow nodes indicate the up-regulated DEGs; purple nodes indicate the down-regulated DEGs. The top 300 DEGs were used for network construction.

To investigate gene expression changes in E13 vs. E19 samples, total RNA from the samples subjected to MeRIP-seq analysis was used to construct the RNA-seq libraries and DEGs were identified using a threshold of *p* value < 0.05 and |log2FC| > 1. This analysis revealed 2880 DEGs in E19 vs. E13. Of these, 1381 were upregulated and 1499 were downregulated (Figure 4A,B, Table S13). Heatmap visualization of the DEGs revealed good repeatability across the three samples in each group and separate clustering of upregulated and downregulated genes (Figure 4C). Next, the DEGs were subjected to enrichment and protein–protein interaction network analysis. GO biological process enrichment analysis showed that the DEGs were significantly enriched in muscle organ development, embryonic skeletal system morphogenesis, and negative regulation of the canonical Wnt signaling pathway (Figure 4D, Table S14). KEGG enrichment analysis of the DEGs revealed that they were significantly enriched in signaling pathways, including the calcium, Wnt, TGF-beta, and melanogenesis pathways (Figure 4E, Table S15). Interestingly, melanogenesis was also enriched in the hypo-methylated peak genes (Table S15). Subsequently, the top 300 most significant DEGs (sorted by *p* value from small to large) were submitted to protein–protein interaction analysis. DEGs were mainly clustered into two parts based on up- and down-regulation, and proteins including myozenin (MYOZ) family members and myomesin 2 (MYOM2), which are associated with skeletal muscle differentiation and muscle fiber fusion, were at the core of the upregulated cluster (Figure 4F). Bone morphogenetic protein (BMP) and fibroblast growth factor receptor (FGFR) families, which regulate the cell cycle and cell proliferation, were at the center of the downregulated cluster (Figure 4F).

### 3.4. Conjoint Analysis of MeRIP-seq and mRNA-seq in E19 and E13

**Figure 5:**
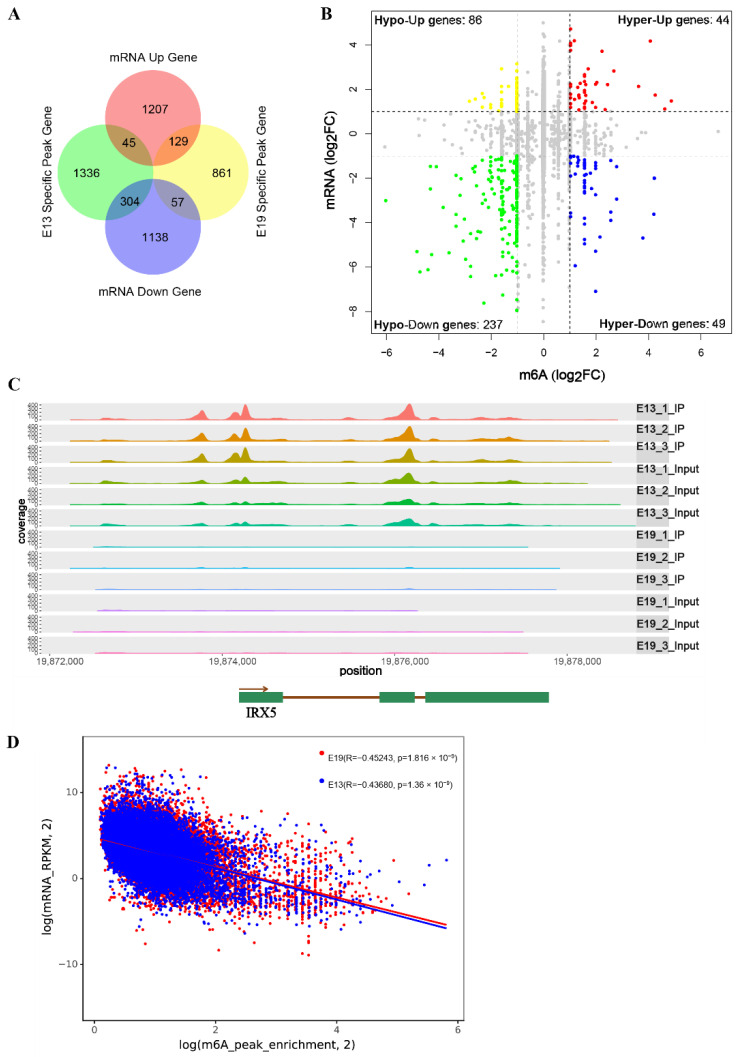
Conjoint analysis of MeRIP-seq and mRNA-seq data. (**A**) Venn diagram of the m6A peaks and DEGs in E19 and E13. (**B**) Distribution of genes with |log2FC| > 1 in both MeRIP-seq and RNA-seq data in E19 and E13. (**C**) The m6A abundances in IRX5 mRNA for the E19 and E13 groups. The m6A peak, which was significantly higher in E19 than in E13, is shown in the black rectangle (*p* < 0.05 and |log2FC| > 1). (**D**) A plot of m6A peak abundance and mRNA expression in E19 and E13.

To investigate the relationship between m6A modification and mRNA expression, m6A peak regions in E19 and E13 samples were cross-compared with the DEGs from RNA-seq data. This analysis identified 129 upregulated and 57 downregulated m6A peak-containing DEGs in E19, and 45 upregulated and 304 downregulated m6A peak-containing DEGs in E13 (Figure 5A, Table S16). In the E19 m6A peak-containing genes, pyruvate dehydrogenase kinase 4 (PDK4) and myosin light chain kinase 2 (MYLK2) are related to muscle differentiation and growth.

Next, dynamically changed peak genes and their expression levels were plotted, and the genes with |log2FC| > 1 in both MeRIP-seq and RNA-seq analyses were screened out (Figure 5B). This analysis revealed that 44 hyper-methylated genes were upregulated (hyper-up genes) and 49 were downregulated (hyper-down genes). For hypo-methylated genes, 86 were up-regulated (hypo-up genes) and 237 were down-regulated (hypo-down genes). These genes were then filtered in the MeRIP-seq and RNA-seq data using *p* < 0.05 as the threshold, and 15 hyper-up, 9 hyper-down, 2 hypo-up, and 58 hypo-down genes with significant differences were obtained (Table 3, Table 4, Table 5 and Table 6 and Table S17). Notably, some genes associated with embryonic development and muscle growth were significantly different in terms of both expression and m6A modification data. The expression and m6A abundance of FBXO40 in E19 samples were significantly higher than in E13 samples. The m6A abundance of iroquois homeobox 5 (IRX5) was significantly elevated, but mRNA levels were significantly reduced (Figure 5C). Pearson analysis of the correlation between m6A modification and expression regulation, using m6A abundance and mRNA expression data, revealed a negative correlation between m6A abundance and the mRNA expression level (E13, R = −0.43680, *p* < 0.01; E19, R = −0.45243, *p* < 0.01), indicating that m6A modification affected gene expression (Figure 5D).

### 3.5. Expression Patterns of the m6A Modification Regulators during Duck Embryonic Skeletal Muscle Development

**Figure 6:**
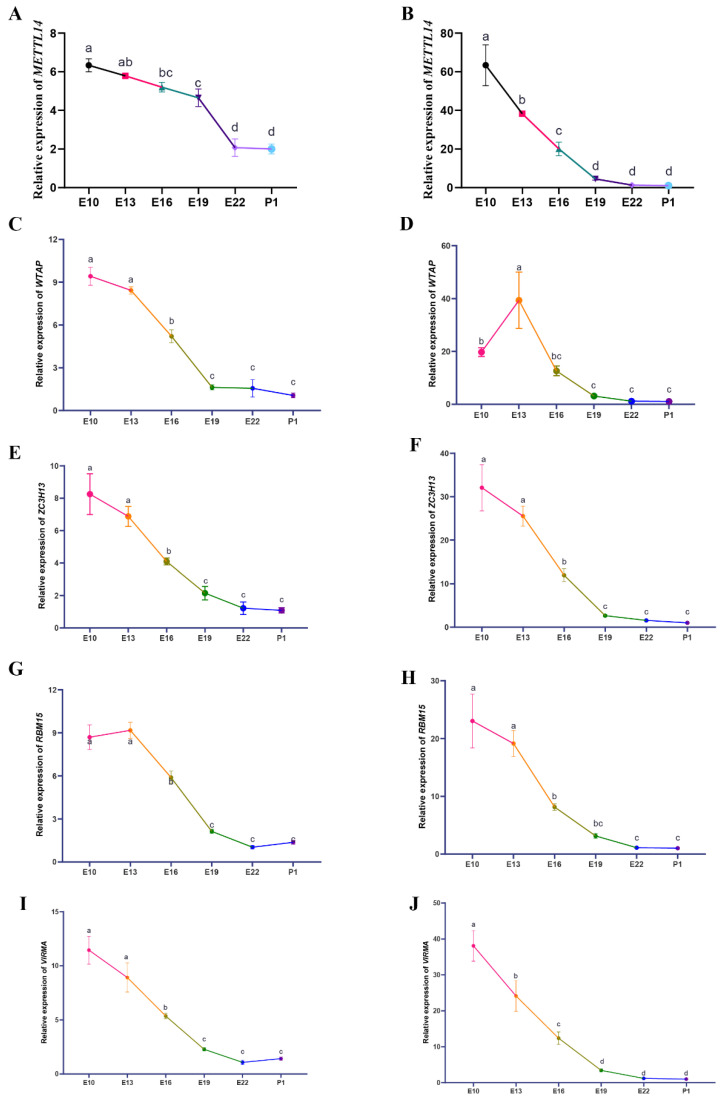
The spatiotemporal expression of *METTL14*, *WTAP*, *ZC3H13*, *RBM15*, and *VIRMA* in E10, E13, E16, E19, E22, and P1 during skeletal muscle development. (**A**,**C**,**E**,**G**,**I**) The expression level of *METTL14* and its cofactors in embryonic breast muscle development. (**B**,**D**,**F**,**H**,**J**) The spatiotemporal expression of m6A-related genes during leg muscle development. In all panels, values are presented as the mean ± S.E.M. Different letters between two groups represent differences that are significant (*p* < 0.05).

**Figure 7:**
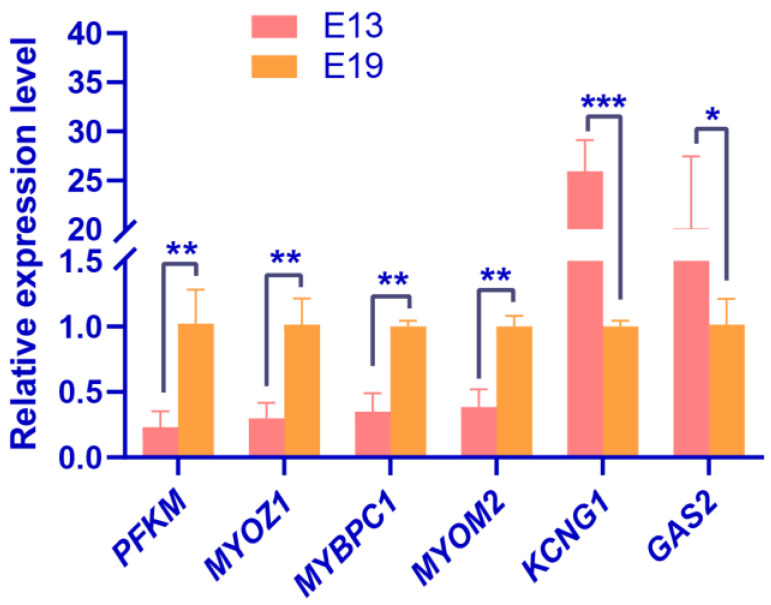
qRT-PCR validation of RNA-seq identification of the DEGs. In all panels, values are presented as the mean ± SEM. * *p* < 0.05; ** *p* < 0.01, *** *p* < 0.001.

Motif sequence analysis showed that m6A modification in duck embryo skeletal muscles may be mediated by METTL14. qRT-PCR analysis of *METTL14* expression during the embryonic skeletal muscle development of duck breast and leg muscles from E10, E13, E16, E19, E22, and 1 day post hatch (P1, 5 samples in each stage) revealed significant changes from E10 to E22 in breast muscles, and marked downregulation from E10 to E19 in leg muscles (Figure 6A,B). In addition, we evaluated the expression levels of *METTL14* cofactors, including *WTAP*, *ZC3H13*, *RBM15*, and *VIRMA* during duck embryonic skeletal muscle development (as above). *ZC3H13*, *RBM15*, and *VIRMA* showed similar downward expression patterns to *METTL14* during embryonic skeletal muscle development (Figure 6E–J). Meanwhile, the expression levels of *WTAP* peaked on E13 during the leg muscle development (Figure 6D), whereas its expression in breast muscle samples showed a downward tendency (Figure 6C). Moreover, we performed qRT-PCR validation for the RNA-seq and the results were consistent with the RNA-seq data (Figure 7). These data showed that *METTL14* and the RNA methyltransferase cofactors’ expression were drastically downregulated from pre-differentiation (before E13) to post-differentiation (after E19) in duck skeletal muscles. We speculate that METTL14-mediated m6A modification plays a crucial role in embryonic skeletal muscle development.

## 4. Discussion

Muscle tissue drives body movement, and animal muscle tissue is a source of high quality protein [27,28]. Muscle development is regulated by many genetic factors, such as DNA methylation genes and non-coding RNAs [29,30]. Epigenetic RNA modification, especially that of m6A, influences various biological processes [9]. Although m6A has been identified in various animals, including pigs, chickens, geese, sheep, cattle, and fish [18,19,20,21,22,25,31,32], it has not been described in ducks, and its role in poultry muscle development is unknown. Here, we selected two important stages of duck embryo skeletal muscle differentiation for MeRIP-seq and found differences in m6A modification patterns before and after skeletal muscle differentiation. Together, our data suggest that m6A methylation plays an important role in duck skeletal muscle development by regulating gene expression.

We found that the m6A modification pattern changed after skeletal muscle differentiation, with a reduction of 1123 m6A peaks and 369 peak genes. More than 73% of the transcripts had one or two m6A peaks and about 13% had ≥ 4 peaks, which is higher than the rates seen in humans, pigs, and chickens [22,33,34]. Motif analysis identified the classical GGACU sequence as the one modified by m6A in duck embryonic skeletal muscle, and METTL3 and METTL14 as potential methyltransferases [11]. However, duck *METTL3* has not been annotated and it is possible that METTL14 mediates m6A modification in duck embryo skeletal muscles. The m6A distribution peaks in duck breast muscle mainly occur in the region before and after the stop codon. This region is proposed to possess transport and translocation features, as well as protein translation regulatory elements, which affect RNA stability, transport, and translocation signal transduction [35]. m6A modification in this region may affect the above regulatory functions by changing RNA conformation. The m6A peak distribution was similar to that observed in pig longissimus dorsi muscles [36], but is different from that seen in chicken abdominal fat tissue [22]. In chicken fat tissue, m6A peaks are mainly distributed before and after the start codon, in the CDS region, and before and after the stop codon. m6A peak distribution patterns may be tissue specific.

Previous studies have shown that different stages of the same tissue have different m6A modification patterns [19,37]. In the E13 and E19 stages of breast muscle tissues, 78.9% of the genes were modified by m6A in both stages, and modification of 21.1% of the genes was stage specific. We speculate that group-specific genes may play important roles in skeletal muscle development. E19-specific m6A genes were significantly enriched in the MAPK signaling pathway. MAPKs are part of a highly conserved network, and the kinases of MAPKs, Mitogen-Activated Protein Kinase 1 (MAPK1), and MAPK3 induce slow fiber-type switching in skeletal muscles [38]. E13 m6A peak-containing genes were mainly enriched in embryo-development- and cell-proliferation-related signaling pathways, including pathways that regulate stem cell pluripotency and cAMP signaling [39,40]. Intersection m6A-containing genes between E19 and E13 were involved in RNA splicing and embryo development. Modifications of the RNA processing genes may affect gene expression, globally affecting mRNA splicing and protein synthesis [41].

Here, 355 differentially methylated peaks (DMPs) and 331 differentially methylated genes (DMGs) were identified. Enrichment analysis revealed that the hyper-DMGs were significantly enriched in AMPK signaling. AMPK induces mitochondrial biogenesis and skeletal muscle glucose uptake during training adaptation, and AMPK signaling mediates muscle-fiber-type transformation [42,43]. Protein–protein interaction network analysis indicated that the DMGs, including FBXO40, SOX2, and DLX5, are involved in skeletal muscle development. Interestingly, the *FBXO40*, *SOX2*, and *DLX5* genes were also differentially expressed, and the differential abundance of m6A is consistent with this expression pattern (Table S13). FBXO40, a member of the F-box protein family, ubiquitinates Insulin Receptor Substrate 1 (IRS1) for degradation, blocks IGF1 signaling, and causes muscle atrophy [44,45]. MeRIP-seq and RNA-seq analyses revealed that, among the m6A peak-associated genes and the hyper-methylated peak genes in E19, many upregulated genes, including *PDK4*, *MYLK2*, and *FBXO40*, are involved in muscle differentiation and growth. Forkhead box O1 (FOXO1) and peroxisome proliferator-activated receptor (PPAR) α/β regulate the PDK4 response to insulin, elevate free fatty acids or hunger-mediated signal transduction, and mediate muscle fiber atrophy [46,47]. *MYLK2* is mainly expressed in striated muscle and mediates the switch of fast and slow muscle fibers in skeletal muscles [48]. We speculate that m6A modification may affect duck muscle development by modulating *PDK4*, *MYLK2* and *FBXO40* expression.

The methyltransferases METTL3 and METTL14 catalyze RNA m6A modification using S-adenosylmethionine as the methylation donor [10,11]. *METTL14* knockout inhibits the differentiation of C2C12 myoblasts and promotes their proliferation [49]. Motif analysis showed that m6A modification in duck embryo skeletal muscle may be mediated by METTL14. The *METTL14* expression level was high before duck embryonic skeletal muscle differentiation (E10 to E16) and decreased significantly between E16 to E19, and remained low at E19 to P1, indicating that *METTL14* influences duck muscle differentiation. We speculate that METTL14 mediates m6A modification in duck embryonic skeletal muscles and regulates muscle development. The underlying mechanism of *METTL14* regulation of skeletal muscle development and the m6A genes induced by METTL14 need to be further explored.

## 5. Conclusions

In summary, we profiled the m6A modification pattern in duck embryonic skeletal muscle, presented the difference between the pre- and post-differentiation m6A patterns in breast muscle, and proposed that m6A modification influences muscle differentiation by regulating gene expression. The current study not only explored the profiling effects of RNA methylation on duck skeletal muscle development, but also laid the groundwork for studying RNA modification in poultry muscle development.

## Figures and Tables

**Table 1:** Summary of MeRIP-seq analysis of E13 and E19 duck breast muscle.

Sample	Raw Reads	Clean Reads	Clean Q30 (%)	Total Mapped (%)	Unique Mapped (%)
E13-1 IP	117,629,262	76,573,096	99	56,802,958 (74.18)	49,117,835 (86.47)
E13-1 Input	120,140,860	72,763,030	99.15	56,820,703 (78.09)	49,836,789 (87.71)
E13-2 IP	147,499,662	97,038,736	99.05	72,227,326 (74.43)	62,036,483 (85.89)
E13-2 Input	108,965,984	64,729,996	99.05	51,040,033 (78.85)	44,598,215 (87.38)
E13-3 IP	128,176,174	83,981,842	99	61,790,574 (73.58)	53,780,759 (87.04)
E13-3 Input	112,164,314	68,385,780	99.1	53,913,372 (78.84)	47,561,820 (88.22)
E19-1 IP	89,938,950	58,507,898	99.05	40,837,436 (69.80)	34,082,169 (83.46)
E19-1 Input	103,104,574	61,018,422	99.1	46,417,242 (76.07)	39,724,873 (85.58)
E19-2 IP	154,264,524	102,036,750	99.05	72,591,319 (71.14)	61,071,036 (84.13)
E19-2 Input	121,768,616	73,999,076	99.1	57,850,115 (78.18)	49,842,110 (86.16)
E19-3 IP	133,140,154	87,204,114	99	60,837,221 (69.76)	51,285,532 (84.30)
E19-3 Input	89,397,936	54,333,714	99.1	41,044,178 (75.54)	35,332,371 (86.08)

**Table 2:** Summary of RNA-seq analysis of E13 and E19 duck breast muscle.

Samples	Raw Reads	Clean Reads	Clean Q30(%)	Total Mapped (%)	Unique Mapped (%)
E13-1	96,829,538	72,084,202	98.7	50,946,411 (81.36)	44,236,015 (86.83)
E13-2	116,560,502	90,806,630	98.7	62,898,538 (80.90)	54,672,390 (86.92)
E13-3	103,815,028	79,869,852	98.7	55,387,445 (80.43)	48,776,577 (88.06)
E19-1	116,673,872	86,389,846	98.65	58,239,780 (79.86)	48,209,452 (82.78)
E19-2	127,745,880	94,700,870	98.75	64,134,736 (79.41)	54,368,577 (84.77)
E19-3	103,438,914	77,876,548	98.65	52,178,329 (78.66)	44,089,921 (84.50)

**Table 3:** List of hyper-up methylated genes that were significantly different between E19 and E13.

Gene ID	mRNA Expression		m6A Level					
	log2FC	*p* Value	Peaks	Chromosome	Peak Start	Peak End	log2FC	*p* Value
LOC101800998	1.4	1.53 × 10^−3^	peak_3641	NC_040047.1	20,800,500	20,802,303	1.9	3.75 × 10^−3^
LOC101797491	1.5	2.81 × 10^−4^	peak_12,651	NC_040059.1	15,312,973	15,313,214	4.9	6.45 × 10^−3^
LOC101794290	2.2	3.57 × 10^−4^	peak_17,441	NC_040074.1	240,177	240,387	2.0	1.85 × 10^−4^
LOC101802407	2.2	3.81 × 10^−7^	peak_18,084	NC_040075.1	22,852,755	22,852,816	2.4	2.25 × 10^−2^
SSUH2	2.8	5.15 × 10^−5^	peak_11,999	NC_040058.1	782,892	782,952	2.7	6.03 × 10^−5^
ITPRID1	1.7	1.08 × 10^−4^	peak_3062	NC_040047.1	59,981,665	59,981,756	4.3	6.08 × 10^−2^
PLCD4	1.6	2.06 × 10^−3^	peak_9384	NC_040052.1	36,291,413	36,291,533	1.2	4.13 × 10^−2^
SFTPA	4.2	2.40 × 10^−6^	peak_8361	NC_040051.1	26,628,000	26,628,150	4.1	1.46 × 10^−4^
SMIM35	3.7	8.85 × 10^−4^	peak_16,504	NC_040070.1	6,803,520	6,803,611	2.2	2.62 × 10^−3^
ARHGEF33	2.1	2.36 × 10^−4^	peak_5261	NC_040048.1	15,704,448	15,704,509	3.6	5.74 × 10^−6^
FBXO40	2.9	1.44 × 10^−6^	peak_636	NC_040046.1	121,465,290	121,465,351	1.7	5.26 × 10^−3^
TTC34	1.1	1.37 × 10^−2^	peak_15,884	NC_040067.1	7,503,028	7,503,089	4.6	6.41 × 10^−31^
TEKT1	1.7	2.55 × 10^−4^	peak_14,752	NC_040065.1	5,379,082	5,379,584	1.3	4.34 × 10^−2^
LOC101801251	2.5	4.96 × 10^−5^	peak_8271	NC_040051.1	16,597,985	16,598,076	11.7	0.00
C2H18orf63	2.3	3.90 × 10^−5^	peak_4010	NC_040047.1	96,440,440	96,440,530	1.4	1.36 × 10^−2^

**Table 4:** List of hyper-down methylated genes that were significantly different between E19 and E13.

Gene ID	mRNA Expression		m6A Level					
	logFC	*p* Value	Peaks	Chromosome	Peak Start	Peak End	logFC	*p* Value
SMOC1	−1.8	6.6 × 10^−6^	peak_8019	NC_040050.1	40,752,605	40,752,666	1.6	4.6 × 10^−3^
LOC101796809	−1.2	8.2 × 10^−4^	peak_6439	NC_040049.1	11,934,318	11,935,468	1.3	2.0 × 10^−2^
LOC113840221	−1.3	4.1 × 10^−3^	peak_18,101	NC_040075.1	25,773,108	25,773,169	1.3	3.6 × 10^−2^
d90837cf-c751-4598-b7b5-9a2a5fe4bd52	−2.0	1.8 × 10^−5^	peak_595 peak_593	NC_040046.1	115,444,079	115,444,140	4.2	1.5 × 10^−26^
PTPRN2	−2.0	6.3 × 10^−4^	peak_3560	NC_040047.1	21,881,949	21,882,982	4.2	2.2 × 10^−39^
GDF7	−2.5	1.1 × 10^−2^	peak_5032	NC_040048.1	108,450,997	108,451,208	1.8	1.1 × 10^−2^
CPA6	−3.6	1.0 × 10^−6^	peak_4114	NC_040047.1	121,248,919	121,249,068	4.2	1.5 × 10^−30^
IRX5	−4.7	3.5 × 10^−7^	peak_11,839	NC_040057.1	19,876,923	19,877,074	3.8	9.8 × 10^−4^
NUAK2	−2.9	3.5 × 10^−6^	peak_16,981	NC_040072.1	4,270,600	4,270,690	2.8	4.2 × 10^−3^

**Table 5:** List of hypo-up methylated genes that were significantly different between E19 and E13.

Gene ID	mRNA Expression		m6A Level					
	logFC	*p* Value	Peaks	Chromosome	Peak Start	Peak End	logFC	*p* Value
MOCOS	1.5	3.8 × 10^−^^4^	peak_4137	NC_040047.1	89,045,565	89,045,745	−2.8	6.6 × 10^−^^7^
LOC101795725	1.6	2.2 × 10^−^^3^	peak_7733	NC_040050.1	40,469,456	40,469,517	−2.3	1.4 × 10^−^^3^

**Table 6:** Top 15 of hypo-down methylated genes that were significantly different between E19 and E13.

Gene ID	mRNA Expression		m6A Level					
	logFC	*p* Value	Peaks	Chromosome	Peak Start	Peak End	logFC	*p* Value
PCLO	−2.0	4.7 × 10^−^^3^	peak_2680	NC_040046.1	193,147,944	193,148,005	−13.9	0.00
VGLL1	−2.3	3.8 × 10^−^^5^	peak_11,203	NC_040055.1	14,011,407	14,011,468	−12.2	0.00
LOC113844117	−4.1	9.4 × 10^−^^6^	peak_9692	NC_040052.1	23,317,486	23,317,607	−7.4	7.7 × 10^−^^287^
PLCH2	−3.0	8.6 × 10^−^^6^	peak_16,762	NC_040067.1	7,693,047	7,693,706	−6.0	1.3 × 10^−^^71^
LOC101801566	−1.5	3.4 × 10^−^^2^	peak_752	NC_040046.1	125,089,939	125,090,030	−4.1	4.5 × 10^−^^20^
CACNA1D	−2.5	2.4 × 10^−^^5^	peak_12,826	NC_040058.1	11,308,422	11,308,543	−4.3	6.1 × 10^−^^15^
FAM81A	−1.5	8.2 × 10^−^^3^	peak_12,390	NC_040057.1	569,354	569,444	−4.3	6.1 × 10^−^^15^
SIM1	−5.4	2.8 × 10^−^^7^	peak_5117	NC_040048.1	74,879,498	74,879,709	−4.3	6.1 × 10^−^^10^
GJB3	−1.7	3.3 × 10^−^^4^	peak_16,909	NC_040069.1	975,274	975,424	−2.6	1.3 × 10^−^^8^
LOC101799119	−5.3	6.0 × 10^−^^5^	peak_3249	NC_040047.1	78,094,137	78,094,228	−4.8	4.7 × 10^−^^8^
FAM83B	−6.2	6.5 × 10^−^^7^	peak_5981	NC_040048.1	92,984,169	92,984,470	−4.7	1.8 × 10^−^^7^
SLC39A8	−3.3	4.4 × 10^−^^6^	peak_6184	NC_040049.1	6,506,530	6,506,653	−3.8	3.9 × 10^−^^7^
LOC101801425	−3.6	9.3 × 10^−^^5^	peak_10,790	NC_040054.1	2,438,445	2,438,536	−3.3	6.5 × 10^−^^7^
HTR1F	−4.2	7.8 × 10^−^^6^	peak_579	NC_040046.1	107,244,996	107,245,206	−3.3	6.5 × 10^−^^7^
AGR2	−5.6	3.4 × 10^−^^5^	peak_3832	NC_040047.1	30,713,854	30,713,913	−3.0	1.7 × 10^−^^6^

## Data Availability

All sequencing data in this study can be found in the NCBI Sequence Read Archive (SRA) (https://submit.ncbi.nlm.nih.gov/, accessed on 22 September 2022) with the accession number PRJNA725663 (MeRIP-seq data) and PRJNA726590 (RNA-seq data).

## Supplementary Materials

The following supporting information can be downloaded at: https://www.mdpi.com/article/10.3390/ani12192593/s1, Figure S1: The correlation coefficient of MeRIP-seq samples; Figure S2: The proportion of 1–2 m6A peaks in each group; Table S1: Information of the qPCR primers; Table S2: the list of intersection peak genes in E13 and E19; Tables S3 and S4: the group-specific peak genes in E13 and E19, respectively; Table S5: the GO biological process analysis with all intersection genes in E13 and E19; Table S6: the KEGG analysis with all intersection genes in E13 and E19; Tables S7 and S8: the KEGG analysis with group-specific peak genes in E13 and E19, respectively; Tables S9 and S10: the differentially methylated peaks (DMPs) and the DMPs associated genes in E19 vs. E13; Tables S11 and S12: the KEGG analysis with the hyper-methylated peak genes and hypo-methylated peak genes in E19 vs. E13, respectively; Table S13: the differentially expressed genes (DEGs) in E19 vs. E13; Table S14: the GO biological process analysis with all DEGs in E19 vs. E13; Table S15: the KEGG analysis with all DEGs in E19 vs. E13; Table S16: the conjoint analysis of group-specific peak genes and DEGs in E19 vs. E13; Table S17: List of hypo-down methylated genes that were significantly different between E19 and E13.
