# Supplementary material for: Profiling Analysis of N6-Methyladenosine mRNA Methylation Reveals Differential m6A Patterns during the Embryonic Skeletal Muscle Development of Ducks
Source: Animals (Basel). 2022 Sep 28;12(19):2593. doi: 10.3390/ani12192593 (PMC9559603; doi:10.3390/ani12192593)
Supplement: Supplementary file 1 [file animals-12-02593-s001.zip › Figure S1.pdf]

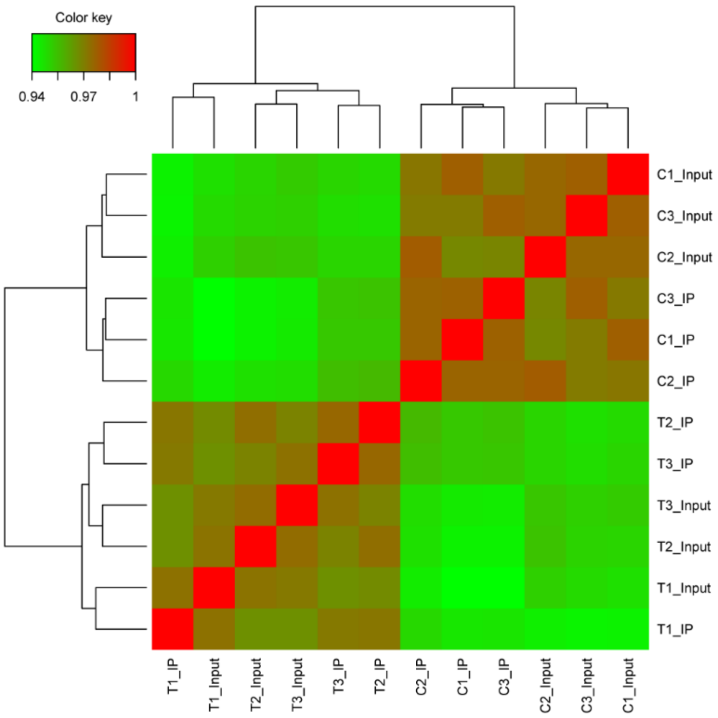

Figure S1. The correlation coefficient of MeRIP-seq samples. C group represents E13 group. T group represents E19 group.
