# Supplementary material for: Profiling Analysis of N6-Methyladenosine mRNA Methylation Reveals Differential m6A Patterns during the Embryonic Skeletal Muscle Development of Ducks
Source: Animals (Basel). 2022 Sep 28;12(19):2593. doi: 10.3390/ani12192593 (PMC9559603; doi:10.3390/ani12192593)
Supplement: Supplementary file 1 [file animals-12-02593-s001.zip › Table S1.pdf]

Table S1. Primers used for qRT-PCR.

Gene Name	Forward Primer (5' to 3')	Reverse Primer (5' to 3')	Annealing Temperature (°C)
METTL14	AACCACCACTGGAAGAATAC	GAAACACAAATGACCTTGGG	58
WTAP	CCGCAGAGTTCACAATGACGAA	CCCTCCAAAGCTTGCACGTA	58
ZC3H13	CCTTCAAGGCACCGATCTCC	TAGTTTCATCCCGCCTTTCGT	58
RBM15	TATGGTAAAGCCACTCCAACCAC	CCGCATCCAAGCTCTCGT	58
VIRMA	CTCCAAGCTATACGCGACCT	CTCGTTTGCACCATCGGACT	58
GAPDH	GGTAGTGAAGGCTGCTGCTGATG	CCACCACACGGTTGCTGTATCC	52-63
PFKM	CGCCGCCTACATCTACGA	GATTCCCTTCCCCTCCTC	60
MYOZ1	GGAACGAGCGATGGGTAT	AGGTATTGGGGTTCTGTTGA	60
MYBPC1	GGCATTTCCAAACCAAGTA	ACAACATAGCCATCTAAACCTG	60
MYOM2	CAAAGAGTCAACGCACAGGT	GCAGATGGGACAACAACAGT	60
KCNG1	TTATCCATCAGCACCATGC	CGGAGGACCAGACCTACTTT	60
GAS2	CTCCTTCGCCCTCACCAA	CCAACACGGACCATCACG	60
